# Novel Psychrophiles and Exopolymers from Permafrost Thaw Lake Sediments

**DOI:** 10.3390/microorganisms8091282

**Published:** 2020-08-22

**Authors:** Ilaria Finore, Adrien Vigneron, Warwick F. Vincent, Luigi Leone, Paola Di Donato, Aniello Schiano Moriello, Barbara Nicolaus, Annarita Poli

**Affiliations:** 1 Consiglio Nazionale delle Ricerche C.N.R., Institute of Biomolecular Chemistry (ICB), via Campi Flegrei 34, 80078 Pozzuoli (Na), Italy; ilaria.finore@icb.cnr.it (I.F.); lleone@icb.cnr.it (L.L.); paola.didonato@uniparthenope.it (P.D.D.); aniello.schianomoriello@icb.cnr.it (A.S.M.); bnicolaus@icb.cnr.it (B.N.); 2 Centre d’études nordiques (CEN) & Département de Biologie, Université Laval, Quebec City, QC G1V 0A6, Canada; avignero@gmail.com (A.V.); warwick.vincent@bio.ulaval.ca (W.F.V.); 3 Department of Science and Technology, University of Naples Parthenope, Centro Direzionale, Isola C4, 80143 Naples, Italy

**Keywords:** bacterial diversity, exopolysaccharides, lake sediments, microbial ecology, permafrost thaw lake, subarctic, psychrophiles, *Pseudomonas*

## Abstract

Thermokarst lakes are one of the most abundant types of microbial ecosystems in the circumpolar North. These shallow basins are formed by the thawing and collapse of ice-rich permafrost, with subsequent filling by snow and ice melt. Until now, permafrost thaw lakes have received little attention for isolation of microorganisms by culture-based analysis. The discovery of novel psychrophiles and their biomolecules makes these extreme environments suitable sources for the isolation of new strains, including for potential biotechnological applications. In this study, samples of bottom sediments were collected from three permafrost thaw lakes in subarctic Québec, Canada. Their diverse microbial communities were characterized by 16S rRNA gene amplicon analysis, and subsamples were cultured for the isolation of bacterial strains. Phenotypic and genetic characterization of the isolates revealed affinities to the genera *Pseudomonas, Paenibacillus, Acinetobacter,* *Staphylococcus* and *Sphingomonas*. The isolates were then evaluated for their production of extracellular enzymes and exopolymers. Enzymes of potential biotechnological interest included α and β-glucosidase, α and β-maltosidase, β-xylosidase and cellobiohydrolase. One isolate, *Pseudomonas extremaustralis* strain 2ASCA, also showed the capability to produce, in the loosely bound cell fraction, a levan-type polysaccharide with a yield of 613 mg/L of culture, suggesting its suitability as a candidate for eco-sustainable alternatives to commercial polymers.

## 1. Introduction

High latitude environments are characterized by persistent cold temperatures, freeze-thaw cycles and extreme seasonality, and these conditions impose severe constraints on biological processes. However, the Polar Regions provide diverse habitats for microbial colonization and growth, including ice-covered seas, perennial snowbanks, glaciers and ice shelves, tundra, polar desert soils, volcanoes and a variety of ice-influenced lakes, rivers and wetlands [1,2]. These extreme environments are increasingly looked to as a source of novel microbes and biomolecules that have potential biotechnological and biomedical applications [3,4]. For example, cold-active enzymes have been identified in microbial cultures from north polar marine environments [5], antibiotic-producing bacteria have been isolated from High Arctic permafrost soils [6], polar glaciers are sites of novel psychrophilic fungi and microalgae [7,8,9], and a variety of exopolysaccharides (EPS) are produced by bacterial isolates from Arctic and Antarctic environments [10,11,12,13]. Many of these EPS compounds have interesting properties, such as metal adsorption, cryoprotection, and emulsifying activities, which suggests that they have potential applications in cosmetic, environmental, and food biotechnological fields as alternatives to the commercial polymers that are currently used [11].

Permafrost landscapes are a typical feature of high latitude environments, and are characterized by the presence of various types of liquid water habitats where microbial communities may grow to high population densities—for example in cold-water springs [14], water tracks beneath the soil surface [15], and ice-covered lakes [16]. One of the most abundant types of waterbody in the Arctic is generated as a consequence of the thawing and collapse of ice-rich permafrost, followed by the filling of these shallow basins by snow and ice melt to produce thermokarst lakes and ponds (‘permafrost thaw lakes’). These waterbodies collectively cover more than 100,000 km^2^ of the northern lands [17], and are capped by ice and snow throughout much of the year. They are known to be transient, microbiological hotspots in the landscape [18], but to date, they have received little attention by microbiologists for culture-based analysis of bacterial isolates.

To our knowledge, this study reports the first attempt to isolate bacterial taxa from the sediments of permafrost thaw lakes, and to characterize their biomolecules, with special attention to exopolymers. These compounds are known to play a major role in low-temperature tolerance and freeze survival of bacteria, and high latitude environments are therefore an excellent set of locations to look for their presence and diversity [13]. We undertook this study at the southern margin of northern permafrost, in subarctic Québec, Canada—where the thawing and collapse of ice-rich permafrost mounds (‘palsas’) produces abundant thaw lakes. The lakes lie in permafrost peatlands, and their waters contain high concentrations of organic matter and diverse planktonic communities of bacteria, archaea and microbial eukaryotes [19,20]. The organic lake sediments are covered by liquid water throughout the year, providing a carbon-rich habitat for continuous growth despite the prolonged winter freeze-up of the surrounding landscape; nevertheless, little is known about their microbial communities. We sampled the bottom sediments from three permafrost thaw lakes during the open water period in summer, and first characterized their sediment bacterial diversity by 16S rRNA gene amplicon analysis to assess the microbial richness of this habitat. With subsamples from the same sediment cores, we then cultured and isolated bacterial strains, and characterized extracellular enzymes and EPS produced by cultures of each isolate.

## 2. Materials and Methods

### 2.1. Sampling

The study sites were located in the Sasapimakwananisikw (SAS) River Valley, in the sporadic permafrost zone of subarctic Quebec, Canada, around 10 km southwest of the village Whapmagoostui-Kuujjuarapik. The valley contains dozens of lakes associated with peatlands and collapsing palsas (a photograph of the valley is given as Figure 7 in reference [21]), and was accessed by helicopter in September 2017. Sediments were sampled from three permafrost thaw ponds (thermokarst lakes) in the valley, identified as SAS2A, SAS2B, and SAS2C. Site physicochemical conditions have been previously described [20]; these black-water lakes are slightly acidic with high concentrations of dissolved organic carbon and anoxic bottom waters. The lakes have high emission rates of methane, and low sulfate concentrations. Sampling was from a Zodiac boat and performed with a MiniGlew sediment coring sampler from the middle of each lake. The sediment material was maintained in the dark at 4 °C prior to subsampling for DNA and culture analyses.

### 2.2. Culture-Independent Approach: Genomic Analysis

To characterize the full bacterial diversity of the benthic microbiome in these peatland thaw lakes, we extracted environmental DNA from the upper cm of the three SAS sediment cores, and analyzed the bacterial 16S rRNA genes by high throughput MiSeq Illumina sequencing. Nucleic acids were extracted from 1g of sediments using the AllPrep DNA/RNA Mini Kit (Qiagen Sciences, Germantown, MD, USA). The V4 region of the 16S rRNA gene was amplified by PCR using the S-D-Bact-0516-a-S-18/S-D-Bact-0907-a-A-20 primer set fused with MiSeq adaptors. PCR reactions were carried out in duplicate in a final volume of 25 μL using Brilliant III Ultra-Fast Q-PCR Master Mix (Agilent, Santa Clara, CA, USA), 0.5 nM of primers and 1 ng of DNA template. The PCR program included 30 cycles of denaturation at 95 °C for 30 s then annealing at 58 °C for 30 s and extension at 72 °C for 1 min. Illumina MiSeq adaptors and barecodes were added during a second PCR step. Each reaction contained 5 μL of GreenGoTaq Reaction Buffer (Promega Corporation, Madison, WI, USA), 0.5 μL of dNTP mix (40 mmol L^–1^ total), 1 μL of each primer (10 μM), 0.125 μL of GoTaq DNA polymerase (Promega), and 1 μL of corresponding PCR1 product. The final volume was adjusted to 25 μL with sterile water. PCR conditions were 95 °C for 2 min; 10 cycles of 95 °C for 40 s, 55 °C for 45 s, and 72 °C for 60 s; and a final extension at 72 °C for 10 min. PCR amplifications were then pooled and purified on agarose gel using the QIAquick Gel Extraction Kit (Qiagen). Libraries were quantified using a QuBit DNA HS Assay Kit (Life Technologies, Carlsbad, CA, USA) and a QuBit 3.0 Fluorometer (Life Tech-nologies) following the manufacturer’s instructions and then paired-end sequenced on an Illumina MiSeq sequencer using a V3 MiSeq sequencing kit (2 × 300 bp) at the Institut de Biologie Intégrative et des Systèmes (IBIS) sequencing platform (Université Laval, Quebec, Canada). Sequences were quality filtered as previously detailed [22] then clustered into operational taxonomic units (OTUs) at 97% of sequence similarity using VSEARCH [23]. The taxonomic affiliations of the OTUs were performed using the Mothur version of the Bayesian classifier with the SILVA 128 database.

### 2.3. Culture-Dependent Approach: Isolation of Psychrophilic Strains

Surface sediment samples (about 1.0 g) were used to inoculate, separately, 30 mL of Tryptone Soya Broth (TSB), 30 mL of medium R2A and 30 mL of medium YN. The temperature of incubation was 10 °C for one week, under slow agitation (40 rpm). The TSB medium contained (g/L): pancreatic digest of casein 17.0; enzymatic digest of soya bean 3.0; NaCl 5.0; K_2_HPO_4_ 2.5; glucose 2.5, pH 7.2. The R2A medium contained (g/L): yeast extract 0.50; proteose peptone 0.50; casamino acids 0.50; glucose 0.50; Na-pyruvate 0.30; K_2_HPO_4_ 0.30; MgSO_4_ 7H_2_O 0.05, pH 7.0. Medium YN contained (g/L): yeast extract 6.0; NaCl 6.0, pH 5.6.

Solid media were obtained by adding 1.8% agar to the compositions described above. After one week of incubation, microbial growth occurred in all tested liquid media, and subsamples were then spread on the corresponding solid media in plates. The colonies were purified by using the serial dilution-plating method at 10 °C, followed by repeated re-streaking on the same solid medium until uniform colonies were obtained.

Genomic DNA was isolated from the cultures using DNAzol (Molecular Research Centre, Inc. Cincinnati, OH, USA) according to the manufacturer’s instruction. PCR amplification and sequencing of the almost full-length 16S rRNA genes of twenty-one strains were obtained from the genomic-DNAs amplification by using universal primers 8F and 1517R of broad specificity in a polymerase chain reaction (PCR). The nucleotide sequence of 16S rRNA genes was analyzed by EzTaxon-e server (https://www.ezbiocloud.net), and the values for pairwise 16S rRNA gene sequence similarity among the closest species were determined using the EzTaxon-e server. A phylogenetic tree was constructed using the software package MEGA X [24] after multiple alignments of the data by CLUSTAL_X [25]. Distances (distance options according to Kimura 2-parameter method) [26] and clustering were based on neighbor-joining [27]. Tree topologies were tested by the bootstrap method of resampling [28] using 1000 replications.

### 2.4. Phenotypic Characterization of Isolates

Cell and colony morphologies were determined by phase contrast microscopy (Nikon Eclipse E400) and by stereomicroscopy (M8, Leica), respectively. Gram reactions were performed by the KOH lysis method, according to Halebian et al. [29], and by testing aminopeptidase activity through commercial strips (Bactident^®^ Merck Millipore, Darmstadt, Germany). Oxidase activity was determined by assessing the oxidation of tetramethyl-*p*-phenylenediamine, and catalase activity by assessing bubble production in a 3% (*v*/*v*) hydrogen peroxide solution. Starch hydrolysis was tested by flooding cultures with Lugol’s iodine solution on solid medium containing 0.2% (*w*/*v*) starch. Xylan and cellulose hydrolysis were tested by flooding cultures with 0.1% Congo Red dye followed by rinsing with 1.0 M NaCl solution on solid medium containing 0.2% (*w*/*v*) xylan and carboxymethyl-cellulose (CMC), respectively.

### 2.5. Exopolymer Production and Recovery

Isolated strains showing mucoid accumulation on agar plates were investigated for exopolymer (EP) production. The bacterial isolates were cultivated in 250 mL of modified buffered broth R2A with reduced complex carbon sources, and enriched by a simple carbon source (1%, *w*/*v*) when their capability was tested, to utilize the selected carbon source and release an exopolymer (EP). Modified buffered medium R2A contained (g/L): yeast extract 0.2, peptone 0.2, casamino acid 0.2, Na-pyruvate 0.3, MgSO_4_ 7H_2_O 0.05 with 0.1 M phosphate buffer pH 7.0 (Na_2_HPO_4_ 7H_2_O 16.35 g/L and NaH_2_PO_4_ 5.4 g/L) plus carbon source 1.0, sterilized by filtration (0.22µm size-pore) and added to the medium R2A after autoclaving. The tested carbon sources were: galactose (Applichem, Darmstadt, Germany), glucose (Sigma-Aldrich, Merck KGaA, Darmstadt, Germany), mannose (Applichem) and sucrose (Sigma-Aldrich).

Bacterial growth was monitored at 10°C under agitation (130 rpm) by measuring the optical density at 540 nm and pH values until the stationary phase was reached (four days of incubation). The cultures were then centrifuged for 30 min at 10,000 rpm, 4 °C. Polymers contained in the extracellular fraction (S) were precipitated by adding 1 volume of cold absolute ethanol, kept at −20 °C overnight and then centrifuged (10,000 rpm, 30 min, 4 °C). The pellet was dissolved in warm distilled water, dialyzed in dialysis tubes (Spectra/Por MWCO; molecular weight cut-off 12–14 kDa) against tap water for 3 days, lyophilized (Heto drywinner) and then weighed.

Cells showed a gelatinous aspect after centrifugation, suggesting that exopolymers could be bound to the cell surface. Procedures to recover the tightly bound (TB) and loosely bound (LB) exopolymer fractions were therefore applied. For recovery of the LB fraction, cells were washed three times with 0.15 M phosphate buffered saline (PBS) pH 7.2 (containing g/L: Na_2_HPO_4_ 1.44, KH_2_PO_4_ 0.24, NaCl 8.0, KCl 0.20), and then centrifuged at 4000× *g* for 40 min at 4 °C. The supernatants were collected and filtered (0.22 µm size-pore) to remove residual cells, dialyzed in tubes (Spectra/Por MWCO; molecular weight cut-off 6–8 kDa) and lyophilized. To recovery the TB fraction, the cells were suspended with 0.1 M NaOH for 4 h at room temperature under agitation, then centrifuged at 20,000× *g* for 20 min at 4 °C to separate the supernatant containing the EP from the residual cell membranes. The supernatant was then dialyzed and lyophilized [11]. EPS, TB and LB fractions were assayed for total carbohydrates according to the phenol-sulfuric acid method [30], for proteins according to Bradford method [31] and uronic acid content according to Blumenkrantz and Asboe-Hansen [32]. All experiments were carried out in duplicate.

### 2.6. Chemical Characterization of Exopolymers

The exopolymer composition was determined after acidic hydrolysis of lyophilized samples (3–4 mg) with 1 mL of 0.5 M trifluoroacetic acid (TFA) at a temperature of 120 °C for 2 h. Monomers were identified by thin layer chromatography (TLC) on silica plates (Silica GelF60, Merck). TLC plates were developed with the following mobile phase: *n*-butanol/acetic acid/water (60/20/20; *v*/*v*/*v*)). The sugar and amino acid fractions were identified by spraying plates with α-naphthol and ninhydrin reagents, respectively [33]. The sugar composition was also identified by high-performance anion-exchange chromatography with pulsed amperometric detection (HPAE-PAD) using a CarboPAC PA1 column and standards for identification and calibration curves [34].

### 2.7. NMR Spectroscopy

^1^H and ^13^C nuclear magnetic resonance (NMR) and heteronuclear single quantum coherence (HSQC) spectra of the exopolysaccharides were performed on a Bruker 600 MHz Bruker spectrometer at 50 °C. For ^1^H analysis, the sample Lev_2ASCA was exchanged twice with D_2_O with an intermediate lyophilizing step and then dissolved in 700 µL D_2_O. Chemical shifts were reported in parts per million, relative to sodium 2,2,3,3-d 4-(trimethylsilyl) propanoate for ^1^H, and CDCl_3_ for ^13^C-NMR spectra [35].

### 2.8. Chromogenic Substrate Hydrolysis: Screening of Glycoside Hydrolase (GH) Activities

Isolates were investigated for the presence of enzymatic activities in the extracellular compartment. For this purpose, 100 mL of cultures in the standard media were collected at stationary phase and centrifuged (20 min at 10,000 rpm, 4 °C). The cell-free supernatants were partially purified with 80% (*w*/*v*) (NH_4_)_2_SO_4_ precipitation and then dialyzed against 50 mM phosphate buffer pH 7.0. The extracellular fractions were assayed for protein content according to Bradford method [29], and tested for glycoside hydrolase activities. The compounds *ortho*-nitrophenyl β-D-galactopyranoside, *o*-nitrophenyl β-D-glucopyranoside, *p*-nitrophenyl β-D-cellobioside, *p*-nitrophenyl β-D-glucopyranoside, *o*-nitrophenyl β-D-cellobioside, *p*-nitrophenyl β-D-lactopyranoside, *p*-nitrophenyl β-D-maltoside, *p*-nitrophenyl β-D-galactopyranoside, *p*-nitrophenyl β-D-xylopyranoside, *p*-nitrophenyl α-D-galactopyranoside, *p*-nitrophenyl α-D-glucopyranoside, *p*-nitrophenyl α-D-maltoside, *p*-nitrophenyl α-L-arabinofuranoside, *p*-nitrophenyl α-L-arabinopyranoside and *o*-nitrophenyl α-D-galactopyranoside were used as colorimetric substrates for β-galactosidase, β-glucosidase, exoglucanase (cellobiohydrolase), β-lactase, β-maltosidase, β-xylosidase, α-galactosidase, α-glucosidase, α-maltosidase, α-arabinofuranosidase and α-arabinopiranosidase enzymatic activities. One hundred μL of 100 mM substrate solution were mixed with 15 μg of proteins in the extracellular fraction and suspended in 50 mM phosphate buffer (1 mL final volume). The reactions were incubated at 37 °C for 1 h. The release of the chromophore *p*-nitrophenol (pNP) from the chromogenic substrates was measured by absorbance at 420 nm. One unit of activity was defined as the activity releasing 1 μmol of *p*-nitrophenol in 1 h under the above assay conditions [36].

## 3. Results

### 3.1. Microbial Diversity of the Thaw Lake Sediments

Sediment samples from the permafrost thaw lakes SAS2A, SAS2B and SAS2C were examined by combining two complementary approaches: Genomic analysis of amplicons and culture-dependent analysis, to assess the microbial diversity of these extreme high-latitude environments, and to identify biomolecules such as enzymes and exopolymers of potential biotechnological interest.

#### 3.1.1. Genomic Analysis

**Figure 1:**
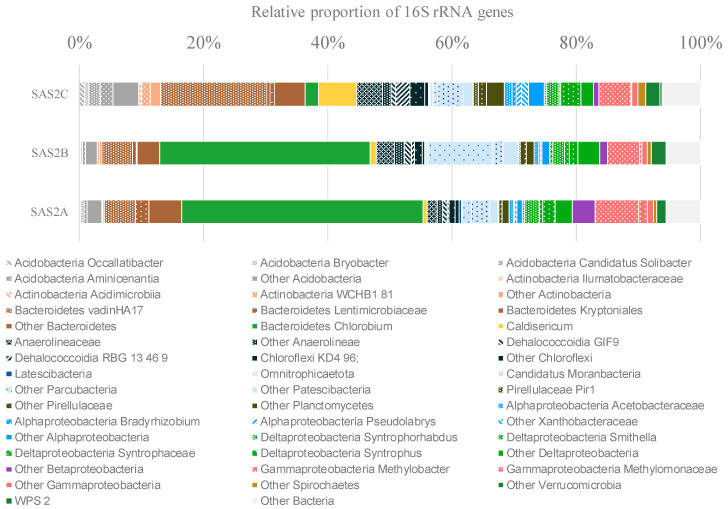
Bacterial community composition determined by Illumina MiSeq 16S rRNA gene sequencing. Only operational taxonomy units (OTUs) with a relative proportion of >1% are shown.

The bacterial community composition of SAS2A, SAS2B and SAS2C sediments was analyzed in detail by 16S rRNA gene sequencing, with an average of 96,800 sequences per sample. The bacterial communities in all three lake sediments had a diverse composition, with up to 1208 different OTUs (at the 97% similarity level). The communities of SAS2A and SAS2B surface sediments were similar and were dominated by *Chlorobium* and Bacteroidetes OTUs, whereas SAS2C sediments showed a reduced proportion of the *Chlorobium* OTUs (Figure 1).

Both aerobic and anaerobic taxa were identified in the sediments. Putative aerobic taxa were represented by members of the Acidobacteria (5.34% of the sequences), Actinobacteria (1.52%), Alpha- (3.66%), Beta- (1.92%) and Gamma-proteobacteria (7.33%), including Methylomonaceae and Pseudomonadaceae families. Anaerobic lineages were mainly represented by *Chlorobium* (24.73%), Deltaproteobacteria (7.94%) and Chloroflexi (8.10% of the sequences). In addition, candidate division bacteria, including Candidatus Moranbacteria, represented a substantial proportion of the sediment community (9.68%) (Figure 1).

Once the cultured isolates were obtained (see below), we re-examined the 16S rRNA gene sequences from the amplicon analysis to determine whether the same taxonomic groups could be detected in the environmental DNA. For the full data set, the genus *Pseudomonas* and the order Sphingomonadales were represented, each contributing up to 0.11% of the reads. *Paenibacillus* accounted for up to 0.007% of the reads, while *Acinetobacter* was not detected. *Staphylococcus* was removed from this analysis, given its presence in a DNA extraction negative control.

#### 3.1.2. Culture-Dependent Analysis

The sediment samples were enriched by using TSB, R2A and YN media for the isolation of psychrophilic bacteria. Several colonies were purified, and twenty-one isolates were obtained. Morphological and biochemical features of isolates are summarized in Table 1. All isolates were rod-shaped except for the strain 2A that showed a cocci shape. Colonies of some isolates, such as strains 2AP, 2CS2, 2CSA, 2CSB, and 2CSC showed fluorescent staining when grown on YN medium; the colonies of strain 2BG were yellow. They were all Gram-positive except for strain 2B that was Gram-negative. They were oxidase positive and most of them showed amylase activity. No strains showed cellulase and xylanase enzymatic activities on agar-plates when grown at a temperature of 10 °C.

Microbial isolates were identified using the EzTaxon-e server (www.ezbiocloud.net/eztaxon), based on the 16S rRNA gene sequence data. Most belonged to the genus *Pseudomonas*; in particular, from SAS2A site five isolates showed the highest similarity to *Pseudomonas extremaustralis* (the similarity ranging from 99.51% to 99.71%) and two strains to *Pseudomonas frederiksbergensis;* other two isolates from SAS2A were most closely related to *Pseudomonas fluorescens;* likewise, from SAS2C site five isolates exhibited highest similarity to *Pseudomonas fluorescens* (similarity ranging from 99.71 to 99.93%). From this latter site, five strains of *Pseudomonas yamanorum,* whose similarity ranged from 99.18 to 99.85%, were also isolated. The isolates also included single strains of the genera *Acinetobacter*, *Staphylococcus*, *Pseudomonas*, *Sphingomonas* and *Paenibacillus*.

**Figure 2:**
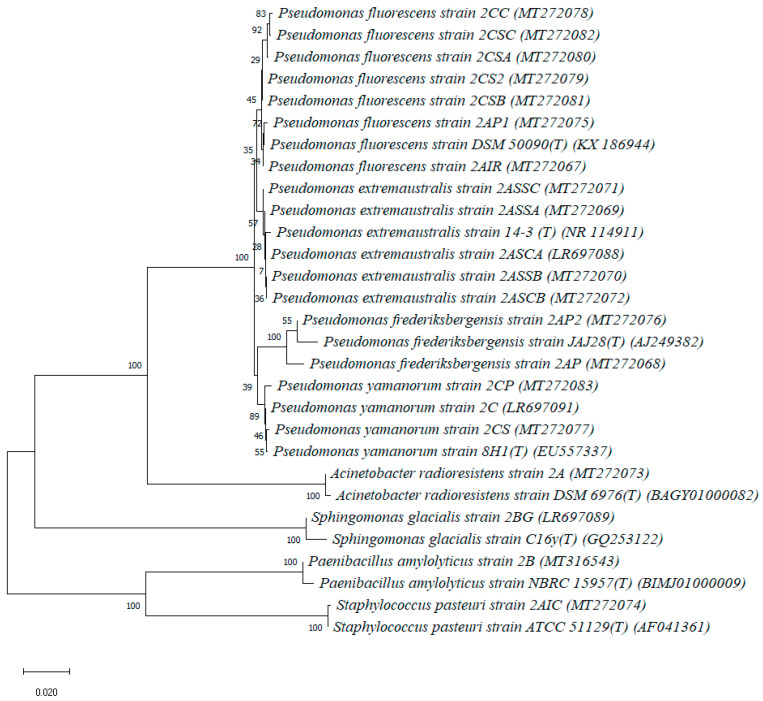
Neighbor-joining tree showing the phylogenetic position of bacterial strains isolated in the present study and the closest relatives based on partial 16S rRNA gene sequences, reconstructed using the Kimura 2-parameter method with 1000 bootstrap replications. The scale represents a genetic distance of 0.02 nucleotide substitutions per site.

The 16S rRNA gene sequences were deposited in GenBank/EMBL/DDBJ under accession numbers as reported in Table 2 and were used for the construction of the neighbor-joining phylogenetic tree (Figure 2). Newly identified isolates were compared to appropriate type species within each genus.

### 3.2. Exopolymer Production and Partial Purification

The isolates with mucoid aspect on agar plates, namely, *Pseudomonas extremaustralis* strain 2ASCA, *Sphingomonas glacialis* strain 2BG, *Paenibacillus amylolyticus* strain 2B and *Pseudomonas yamanorum* strain 2C, were grown on simple carbon sources, such as glucose, galactose, mannose and sucrose in minimal growth media, with the aim to evaluate the exopolymer production capability. *Sphingomonas glacialis* strain 2BG was able to utilize only galactose and glucose for growth, reaching an optical density at 540 nm of 1.054 and 1.018, respectively, after four days of incubation at a temperature of 10 °C; only a small amount of exoproduct (about 55 mg/L) was recovered into the supernatants from each sugar tested, and no carbohydrate polymers were detected. Similarly, *Pseudomonas yamanorum* strain 2C grew on galactose, glucose, and mannose as main carbon sources, reaching an optical density at 540 nm of 1.150, 1.181, and 1.079, respectively, after four days of incubation at a temperature of 10 °C; a small amount of exoproduct (15–26 mg/L) was recovered from the supernatants when the strain was grown in the presence of the tested sugars tested, and no carbohydrate polymers were detected. *Paenibacillus amylolyticus* strain 2B and *Pseudomonas extremaustralis* strain 2ASCA showed good growth on all four sugar sources tested with an O.D. at 540 nm > 1.000 (Table 3).

Analysis of the three purified fractions, extracellular (S), tightly bound (TB) and loosely bound (LB) fractions, revealed that *Paenibacillus amylolyticus* strain 2B and *Pseudomonas extremaustralis* strain 2ASCA were able to synthesize exopolymers. Strain 2B produced an exoproduct showing both a proteic and saccharidic nature, in different ratios, when it was grown in mR2A medium plus galactose or plus sucrose as main carbon sources, at 10 °C under agitation; these products, named “P_2BG” and “P_2BS” were both located in the tightly bound cell fraction and showed a yield of 6.76 and 23 mg/L, respectively (Table 3). Thin layer chromatography analyses of these products, carried out after acidic hydrolysis reactions, revealed the presence of two monomers giving a positive ninhydrine reaction and showing the retention factor of glutamic acid and glutamine for P_2BG. The hydrolyzed P_2BS exhibited on TLC four bands positive for ninhydrine color reaction with the same retention factor of phenylalanine, glutamic acid, galactosamine and glutamine (data not showed).

Strain 2ASCA produced an exopolysaccharide when it was grown in mR2A medium plus sucrose as sole carbon source, at temperature of 10°C under agitation; this exopolymer, named “Lev_2ASCA” was located in the loosely bound cell fraction and it was produced with a yield of 613.2 mg/L, of which almost all was polysaccharide (Table 3). Preliminary studies of the Lev_2ASCA polymer were carried out by analyzing the monomer composition by TLC after acidic hydrolysis, and Lev_2ASCA polymer was found to be composed essentially of fructose units. This composition was confirmed by HPLC-Dionex analysis. The chromatogram showed a main peak with a retention time typical of the fructose standard (15.68 min), indicating that Lev_2ASCA has a sugar composition consisting of fructose residues (data not shown).

### 3.3. NMR Spectroscopy

**Figure 3:**
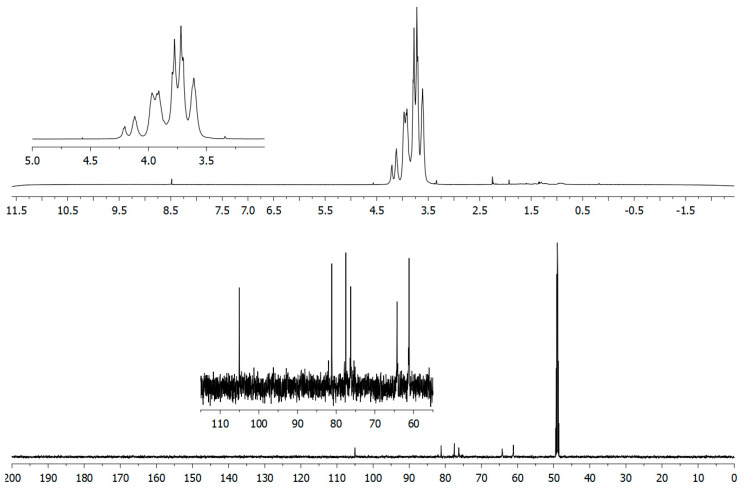
1D-NMR analysis of the Lev_2ASCA polymer: ^1^H (upper) and ^13^C (lower) traces were recorded with a 600 MHz Bruker spectrometer. The x-axis for each spectrum were in chemical shifts reported as parts per million (ppm) with reference to D_2_O and to CD_3_OD for ^1^H and ^13^C spectrum, respectively.

**Figure 4:**
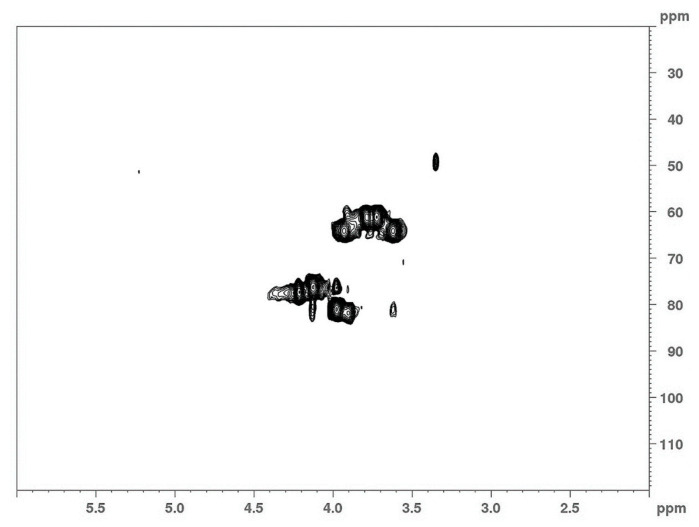
2D-NMR analysis of the Lev_2ASCA polymer: The HSQC spectrum was recorded with a 600 MHz Bruker spectrometer.

Structural analysis of the Lev_2ASCA polymer was performed by means of NMR. The comparison between 1D (Figure 3) and 2D (Figure 4) spectra confirmed that the isolated polymer is a levan type polysaccharide. In the ^1^H spectrum (upper trace in Figure 3), the main signals were found in the bulk region were the ring protons resonate; i.e., at 4.21 ppm, 4.12 ppm, 3.97 ppm, 3.92 ppm, 3.78 ppm, 3.72 ppm and 3.61 ppm, which is typical of the furanose ring of a levan polymer (Poli et al., 2009). The ^13^C spectrum (lower trace in Figure 3) also showed the typical signals of a levan, with resonances at 105.5 ppm, 81.15 ppm, 77.51ppm, 76.23 ppm, 64.24 ppm and 61.19 ppm. The analysis of the HSQC spectrum (Figure 4) showed main cross peaks at 81.15 ppm/3.97 ppm (C5/H5), 77.51 ppm/4.21 ppm (C3/H3), 76.23 ppm/4.12 ppm (C4/H4), 64.24 ppm/3.92 and 3.61 ppm (C6/H6a,b), 61.19 ppm/3.78 and 3.72 ppm (C1/H1a,b).

### 3.4. Chromogenic Substrate Hydrolyses: Screening of Glycoside Hydrolase (GH) Activities

Some isolates, namely, *Pseudomonas extremaustralis* strains 2ASSA and 2ASCA, *Acinetobacter radioresistens* strain 2A, *Paenibacillus amylolyticus* strain 2B, *Sphingomonas glacialis* strain 2BG, *Pseudomonas fluorescens* strains 2CSB and 2CS2 and *Pseudomonas yamanorum* strain 2C were investigated for the presence of glycoside hydrolase activities in the cell free supernatant, given that species belonging to the same genera are described in the literature as being capable of producing glycoside hydrolase activities (Table 4).

For this purpose, nitrophenyl glycosides were utilized as substrates for enzymatic assays at 37 °C. After 1 h, all tested microorganisms exhibited β-galactosidase and glucosidase activities when a pyranose ring form of substrate was used, while analogous substrates in which the saccharidic moieties were in furanose forms were not hydrolyzed. In addition, *Pseudomonas extremaustralis* strains 2ASSA and 2ASCA also released an α-glucosidase enzyme, with activities almost nine-times higher than the beta form. *Pseudomonas fluorescens* strain 2CSB and *Pseudomonas yamanorum* strain 2C expressed a β-glucosidase hydrolysis function that exclusively attacked bonds in the *ortho* position. *Paenibacillus amylolyticus* strain 2A displayed a versatile maltosidase for α and β linkages, while *Pseudomonas extremaustralis* strain 2ASCA had only a week α-maltosidase, unlike *Pseudomonas yamanorum* strain 2C and *Pseudomonas fluorescens* strain 2CS2 that exhibited strong β-maltosidase activity. All tested isolates showed β-xylosidase activity and did not produce β-lactase; in addition, they were able to hydrolyze β bonds linking cellobiose units in the para, but not ortho, position.

## 4. Discussion

Permafrost thaw lakes are abundant habitats for microbial life, yet their sediment microbial diversity has been little explored. In this study, we examined the microbial communities of sediments collected in three sites in the rapidly thawing southern margin of permafrost, in subarctic Québec, Canada. The diverse microbial communities inhabiting the thaw lake sediments were analyzed by means of next-generation sequencing of 16S rRNA gene amplicons from environmental DNA, and Sanger sequencing of the 16S rRNA genes in cultured psychrophilic isolates. Based on the literature data and their observed phenotypic features, bacterial isolates were selected to investigate their potential as producers of extracellular biomolecules.

The Sanger sequencing of cultures allowed us to identify 21 diverse isolates, mostly Gammaproteobacteria (86%), but also Bacilli (9.5%) and Alphaproteobacteria (4.5%). In particular, 18 isolates were in the genus *Pseudomonas*, consistent with the detection of *Pseudomonas* in the environmental DNA analysis. Other taxa detected by the amplicon analysis were likely precluded by the culture conditions chosen here, such as the designated temperature, pH, sodium chloride content, organic matter, oxygen availability and absence of light. These conditions selected for specific chemoorganoheterotrophs and would have excluded anaerobes such as *Chlorobium, Deltaproteobacteria* and *Chloroflexi* that were well represented in the amplicon sequences. The isolates accounted for only a small portion of the total microbial diversity of the thaw lake sediments, as expected, and were rare taxa that proliferated in culture.

Cold-dwelling microbes isolated from the low-temperature habitats are known to release biomolecules of broad interest in several biotechnological sectors [12,37,38,39,40,41,42]. In this study, the cultured isolates were investigated for the production of exopolymers and extracellular enzymes by means of glycoside hydrolase activities. *Pseudomonas extremaustralis* strain 2ASCA, isolated from the sample SAS2A, produced the levan-type exopolymer Lev_2ASCA. This compound was recovered from the loosely bound cell fraction when the bacterium was grown at 10 °C in the presence of sucrose as main carbon source. Levan is the homopolymer of fructose and is known to be synthesized as an exopolysaccharide by many bacterial genera, including *Acetobacter*, *Azotobacter*, *Bacillus*, *Corynebacterium*, *Gluconobacter*, *Halomonas, Mycobacterium*, *Pseudomonas*, *Streptococcus*, and *Zymomonas* [43]. Studies have drawn attention to the wide potential of levan for its anti-cancer, anti-bacterial and anti-viral activities, in addition to its capabilities to enhance calcium absorption, to decrease plasma glucose content in diabetic rats, to inhibit metal corrosion, to aid drug delivery and to act as an adhesive [44].

Levan production by strain 2ASCA had a novel feature in that the polymer was cell-associated and not dispersed in the supernatant. Therefore, polymer recovery was simplified and faster relative to other exopolysaccharide producers, since by means of only a single centrifuge step, all polymers were precipitated by forming a gel layer above the cellular pellet. An important aspect of this biochemical isolation procedure is that it did not require the use of organic solvents. This raises the value of levan from *Pseudomonas extremaustralis* strain 2ASCA, since it would lend itself to eco-friendly production systems, without the need to use organic chemicals that create waste disposal problems. The isolate *Paenibacillus amylolyticus* strain 2B from the SAS2B sediments, produced a biofilm with both protein and saccharide constituents when grown in the presence of galactose or sucrose as main carbon sources at 10 °C. These products were also located in the tightly bound cell fraction, which may similarly facilitate biochemical extraction of exopolymers from this isolate. Further studies are needed to more completely understand the chemical composition of these exoproducts, and their role in permafrost microbial ecosystems.

Although the optimization of enzyme expression was not the focus of this study, our preliminary results show levels of activity that are consistent with those reported in the literature [45,46,47,48,49]. Under the tested conditions, the most promising activities were of α-glucosidase from *Pseudomonas extremaustralis* strains 2ASSA and 2ASCA, and of β-galactosidase, β-glucosidase β-xylosidase and cellobiohydrolase from several isolates. These results encourage future studies to purify these glycoside hydrolase enzymes, to identify their exact cellular localization and to assess the culture and substrate conditions for their maximum expression.

The results of this study are consistent with the view that little-explored areas of our planet such as extreme Arctic environments contain high microbial biodiversity, with a level of genomic and metabolomic richness that has been largely unrecognized to date [50]. As shown here for permafrost thaw lake sediments, these habitats contain microbiota that can produce a wide array of interesting biomolecules, including enzymes and extracellular polysaccharides. These bacterial biomolecules offer potential for eco-sustainable biotechnology applications.

This study delivered insights into the microbial diversity of communities present in the sediments collected in three permafrost thaw lakes in subarctic Québec, Canada, using both culture-dependent and -independent approaches. Next Generation Sequencing provides technologies complementary to conventional isolation, amplification and Sanger sequencing of 16S rRNA genes for bacterial identification, especially in case of complex samples, because they provide rapid information, avoid any preliminary culturing step, and permit classification to family and genus levels with accuracy. At the species level, however, there are limitations due to the restricted discriminatory capacity of the 16S rRNA gene for rare taxa, thereby underestimating the full bacterial diversity. Parallel use of both procedures is desirable for obtaining complementary information about the taxonomic composition, genetic richness, and potential metabolic pathways of microbial communities [51]. On the one hand, the cultivable microbial populations represent only a minute fraction of the whole community, and are strictly limited to the growing parameters set up by the microbiologist. However, on the other hand, culture-based methods remain indispensable for the recovery and characterization of biomolecules synthesized by microorganisms, and for studying microbial adaptation mechanisms and ecological roles. Only through a culture-dependent approach is it possible to access and fully characterize the wide range of microbial metabolic products that are of interest in biotechnology.

## Figures and Tables

**Table 1:** Main properties of strains isolated from SAS2A, SAS2B, and SAS2C sampled sites.

Sample	Medium	Isolate Name	Colony Morphology	Catalase	Oxidase	Amylase
SAS2A	TSB	2AIR	cream opaque, irregular edges	+	+	+
	TSB	2AP	beige, irregular edges	+	+	
	TSB	2ASSA	cream, small, irregular edges	+	+	W
	TSB	2ASSB	cream, irregular edges	+	+	+
	TSB	2ASSC	pale yellow, small, irregular edges	+	+	+
	TSB	2ASCA	cream, irregular edges	+	+	W
	TSB	2ASCB	pale yellow, irregular edges	+	+	+
	YN	2A	cream, small irregular edges	+	+	-
	YN	2AIC	cream, irregular edges	W	+	+
	YN	2AP1	fluorescent yellow, small and regular edges	+	+	-
	R2A	2AP2	cream, small and regular edges	-	+	-
SAS2B	R2A	2BG	yellow, small and regular edges	-	+	-
	TSB	2B	cream, small and regular edges	-	+	-
SAS2C	TSB	2CS	beige, regular edges	-	+	W
	TSB	2CC	beige, irregular edges	+	+	+
	YN	2CS2	fluorescent yellow, irregular edges	-	+	-
	YN	2CSA	fluorescent yellow, irregular edges	W	+	+
	YN	2CSB	fluorescent yellow, small and regular edges	W	+	+
	YN	2CSC	fluorescent yellow, regular edges	-	+	W
	R2A	2CP	cream, small and regular edges	-	+	+
	R2A	2C	white shiny, small and regular edges	W	+	W

+ = positive response; - = negative response; W = weak response.

**Table 2:** Identification of isolates by 16S rRNA gene sequencing.

Sample	Isolate Name	16S rRNA (bp)	Accession Number	16S rRNA Gene Similarity (%)
SAS2A	2AIR	1375	MT272067	*Pseudomonas fluorescens* 100%
	2AP1	1419	MT272075	*Pseudomonas fluorescens* 99.79%
	2AP	1389	MT272068	*Pseudomonas frederiksbergensis* 98.69%
	2AP2	1360	MT272076	*Pseudomonas frederiksbergensis* 99.77%
	2ASSA	1393	MT272069	*Pseudomonas extremaustralis* 99.70%
	2ASSB	1389	MT272070	*Pseudomonas extremaustralis* 99.71%
	2ASSC	1369	MT272071	*Pseudomonas extremaustralis* 99.70%
	2ASCA	1386	LR697088	*Pseudomonas extremaustralis* 99.55%
	2ASCB	1423	MT272072	*Pseudomonas extremaustralis* 99.51%
	2A	1396	MT272073	*Acinetobacter radioresistens* 99.93%
	2AIC	1432	MT272074	*Staphylococcus pasteuri* 99.85%
SAS2B	2BG	1331	LR697089	*Sphingomonas glacialis* 99.62%
	2B	1405	MT316543	*Paenibacillus amylolyticus* 100%
SAS2C	2CC	1383	MT272078	*Pseudomonas fluorescens* 99.71%
	2CS2	1409	MT272079	*Pseudomonas fluorescens* 99.85%
	2CSA	1379	MT272080	*Pseudomonas fluorescens* 99.71%
	2CSB	1403	MT272081	*Pseudomonas fluorescens* 99.85%
	2CSC	1409	MT272082	*Pseudomonas fluorescens* 99.93%
	2CP	1367	MT272083	*Pseudomonas yamanorum* 99.18%
	2CS	1398	MT272077	*Pseudomonas yamanorum* 99.78%
	2C	1392	LR697091	*Pseudomonas yamanorum* 99.85%

**Table 3:** Growth and exopolymer yields of *Paenibacillus amylolyticus* strain 2B and *Pseudomonas extremaustralis* strain 2ASCA in the presence of single carbon sources.

Microorganism	Carbon Sources	O.D.* at λ540 nm	Fraction Yield: mg EP/L	Carbohydrate Content (%. *w*/*w*)	Protein Content (%. *w*/*w*)
*Paenibacillus amylolyticus* strain 2B	galactose	1.490	S: 68	0.0	1.4
			LB: 30.4	1.5	2.83
			TB: 113.2	21.2	5.98
	sucrose	1.232	S: 184	12	0.69
			LB: 40.4	8.9	5.21
			TB: 123.2	11.1	18.63
*Pseudomonas extremaustralis* strain 2ASCA	galactose	1.054	S: 167.6	2	0.23
			LB: 28.8	2.6	0.75
			TB: 47.2	26.6	3.3
	glucose	1.246	S: 275.2	1.1	0.52
			LB: 51.2	6	0.63
			TB: 77.6	12.13	3.23
	mannose	1.061	S: 135.2	3.3	0.6
			LB: 27.6	2.8	0.7
			TB: 21.2	25	3.4
	sucrose	1.430	S: 158.4	3.99	0.63
			LB: 613.2	86.02	0.15
			TB: 34.8	18.65	3.53

All values are averages of two determinations. Uronic acid content in all exoproducts ranged from 0.02 to 2.0% *w*/*w*. * Optical density measured after four days of incubation at 10 °C under agitation (130 rpm). S: polymer in extracellular fraction. LB: polymer in loosely bound fraction. TB: polymer in tightly bound fraction.

**Table 4:** Glycoside hydrolase (GH) activities detected through nitrophenyl (NP) glycosides in the extracellular compartment of selected isolates.

	(NP) Glycosides *														
Strain	1	2	3	4	5	6	7	8	9	10	11	12	13	14	15
*P. extremaustralis* strain 2ASSA	27	202	8	2	836	122	30	8	24	46	8	569	591	9	5
*A. radioresistens* strain 2A	2	192	19	7	75	109	26	5	6	16	13	634	529	10	5
*P. extremaustralis* strain 2ASCA	42	248	27	3	945	130	41	75	7	20	171	640	62	9	7
*P. amylolyticus* strain 2B	31	239	7	5	57	119	42	106	204	33	22	608	640	9	7
*S. glacialis* strain 2BG	10	185	3	6	13	363	38	13	5	951	3	533	353	4	6
*P. fluorescens* strains 2CSB	19	199	18	7	7	589	205	6	8	79	16	539	111	6	7
*P. yamanorum* strain 2C	4	232	3	9	3	117	263	3	181	25	3	189	106	5	10
*P. fluorescens* strains 2CS2	29	286	11	57	6	111	29	1	180	19	5	512	603	6	4

Units of GH activities, expressed as µmol of pNP released under the assay conditions. * 1 = *p*-nitrophenyl α-d-galactopyranoside; 2 = *p*-nitrophenyl β-d-galactopyranoside; 3 = *o*-nitrophenyl α-d-galactopyranoside; 4 = *o*-nitrophenyl β-d-galactopyranoside; 5 = *p*-nitrophenyl α-d-glucopyranoside; 6 = *p*-nitrophenyl β-d-glucopyranoside; 7 = *o*-nitrophenyl β-d-glucopyranoside, 8 = *p*-nitrophenyl α-d-maltoside; 9 = *p*-nitrophenyl β-d-maltoside; 10 = *p*-nitrophenyl α-l-arabinofuranoside; 11 = *p*-nitrophenyl α-l-arabinopyranoside; 12 = *p*-nitrophenyl β-d-xylopyranoside; 13 = *p*-nitrophenyl β-d-cellobioside; 14 = *o*-nitrophenyl β-d-cellobioside; 15 = *p*-nitrophenyl β-d-lactopyranoside. All values are averages of two determinations.
